# Gender-specific influence of socioeconomic status on the prevalence of migraine and tension-type headache: the results from the Korean headache survey

**DOI:** 10.1186/1129-2377-14-82

**Published:** 2013-10-04

**Authors:** Min Kyung Chu, Dong-Wook Kim, Byung-Kun Kim, Jae-Moon Kim, Tae-Won Jang, Jeong Wook Park, Kwang Soo Lee, Soo-Jin Cho

**Affiliations:** 1 Department of Neurology, Hallym University Sacred Heart Hospital, Hallym University College of Medicine, Anyang, Republic of Korea; 2 Department of Neurology, Dongtan Sacred Heart Hospital, Hallym University College of Medicine, 40, Seokwoo-dong, Hwaseong-si, Gyeonggi-do 445-170, Republic of Korea; 3 Department of Neurology, Eulji University School of Medicine, Seoul, Republic of Korea; 4 Department of Neurology, Chungnam National University, College of Medicine, Daejeon, Republic of Korea; 5 Department of Occupational & Environmental Medicine, Seoul St.Mary’s Hospital, the Catholic University of Korea, Seoul, Republic of Korea; 6 Department of Neurology, Uijeongbu St.Mary’s Hospital, the Catholic University of Korea, Uijeongbu, Republic of Korea; 7 Department of Neurology, Seoul St.Mary’s Hospital, the Catholic University of Korea, Seoul, Republic of Korea

**Keywords:** Socioeconomic status, Prevalence, Migraine, Tension-type headache, Gender, Education

## Abstract

**Background:**

Socioeconomic status plays an important role in pain coping strategy. Its influence on migraine and tension-type headache may differ by gender. This study aimed to evaluate how socioeconomic status affects the prevalence of migraine and tension-type headache by gender.

**Methods:**

We used data from the Korean Headache Survey, a population-based sample of Koreans aged 19–69 years. Education level, district size, and household income were evaluated as socioeconomic variables.

**Results:**

Among 1507 participants, the 1-year prevalence rates of migraine and tension-type headache were 8.7% [95% confidence interval (CI) 1.9-4.6%] and 29.1% (95% CI 25.7-32.5%) in women and 3.2% (95% CI 1.9-4.6%) and 32.5% (95% CI 29.1-35.9%) in men, respectively. In women, multiple regression analysis found that living in rural areas was related to higher prevalence of migraine [odds ratio (OR) 4.52, 95% CI 1.85-11.02] and lower prevalence of tension-type headache (OR 0.29, 95% CI 0.15–0.58) and college-level education was related to lower prevalence of tension-type headache (OR 0.37, 95% CI 0.18–0.74). In men, multiple regression analysis failed to reveal significant influences of any socioeconomic variable on the prevalence of migraine or tension-type headache.

**Conclusions:**

The influence of socioeconomic status on migraine and tension-type headache differs by gender, with women being more susceptible to socioeconomic influence.

## Background

Socioeconomic status (SES), an economic and sociological combined variable which represents individuals position or status individuals hold within the structure of society, is consistently associated with morbidity, life expectancy and risk of pain [1,2]. However, previous studies about the influence of SES on migraine or tension-type headache (TTH) were inconclusive [3-6]. These discrepancies may be related to cultural aspects of society or design of the study.

Primary headache disorders, including migraine, have notable gender differences in prevalence [7,8], which are mainly rooted in differences in sex hormones, but women’s social disadvantage may also play a role [9]. SES has been found to have an important role in pain coping strategy, so gender inequalities may influence the prevalence of migraine or TTH [1]. In addition, previous studies on the influence of SES on primary headache disorders have been conducted in Europe or the USA, but rare in Asian countries.

In the present study, we aimed to evaluate the gender specific influences of SES on the prevalence of primary headache, especially migraine and TTH, using nationwide data from the Korean Headache Survey (KHS) [10,11].

## Methods

### Study population and sampling method

KHS was a population-based, nationwide, cross-sectional survey conducted in March 2009 to determine the status of primary headache disorders among adults aged 19–69 years. The details about KHS have been published elsewhere [10,11] and brief descriptions are followed. This study included all Korean territories except Jeju-do and was conducted with the technical support of Gallup Korea, a global social research company. Korea is geographically scattered into 15 administrative divisions ('do’) except Jeju-do, and each administrative division is further divided into the 60 basic administrative units ('si’, 'gun’ or 'gu’). The estimated population of Korea in 2009 was 49,759,141 individuals according to data from the National Statistical Office, of which approximately 34,782,714 people were aged 19 years or over [12].

We planned to sample 1500 individuals based on the population structure and a 2-stage systematic random sampling method was adopted. The 15 administrative divisions were designated as the primary sampling units and appropriate sample numbers were assigned at each primary sampling unit according to the population distribution. In the second stage, 60 representative basic administrative units were selected, in each of which we assigned a target sampling number regarding age, gender, and occupation. The estimated sampling error of our study was ±2.5%, with a 95% confidence interval.

Finally face-to-face interviews were conducted with 1507 participants; these were conducted by trained interviewers based on a structured questionnaire. The questionnaire included demographic variables, a headache profile and the Headache Impact Test-6 (HIT-6) questionnaire, which was designed to evaluate headache-related burden [13]. We informed survey topic to candidates as “social health issue”, rather than “headache disorder” before acceptance of survey to minimize interest bias of our survey. To assess the validity of migraine and TTH diagnoses by comparing them with those of neurologists, we asked all participants whether they would agree to an additional telephone interview with neurologists at initial interview. Overall, 135 of the 1507 participants agreed to an additional telephone interview for validation of their headache diagnosis, and 82 of them completed the telephone interview with nine eight neurologists and one dentist (see acknowledgements) within 2 weeks of the initial face-to-face interview.

Diagnosis of migraine was based on the assigned A to D criteria for migraine without aura in the ICHD-II [A: five or more attacks fulfilling criteria B-D; B: attack duration of four to 72 hours; C: any two of the four typical headache characteristics (i.e., unilateral pain, pulsating quality, moderate-to-severe intensity, and aggravation by routine physical activity); D: attacks associated with at least one of the following conditions (nausea or vomiting, or both photophobia and phonophobia)]. The estimated sensitivity and specificity for migraine were 75.0% and 88.2%, respectively [11,14].

Diagnosis of TTH was based on the assigned A to D criteria for TTH in the ICHD-2 [A: Ten or more attacks fulfilling criteria B-D; B: attack duration of 30 minutes to 7 days; C: any two of the four typical headache characteristics (i.e., Bilateral pain, pressing/tightening quality, mild to moderate intensity, and not aggravation by routine physical activity); D: attacks associated with both following conditions (no nausea or vomiting, no more than one of photophobia and phonophobia)]. For TTH, the estimated sensitivity and specificity were 86.2% and 75.5%, respectively [11,14].

### Assessment of socioeconomic status

Education level, district size and household income were evaluated as socioeconomic variables. We classified seven 'si’ areas (Seoul, Busan, Daegu, Incheon, Gwangju, Daejeon, and Ulsan) as 'large city,’ other 'si’ areas as 'medium-to-small city,’ and 'gun’ areas as 'rural areas’ for our analysis. Education levels were classified into college or more (> 12 years), high school (9–12 years) and middle school or below (< 9 years). Monthly household income was arbitrarily classified as below 1.99 million Korean won (KRW), 2.00–2.99 million KRW, 3.00–3.99 million KRW, and more than 4.00 million KRW.

### Statistical analysis

Statistical analyses were performed separately for all women, men, and participants. The weighted prevalence of migraine and TTH were calculated with 95% confidence interval. The odds ratios of variables including SES were estimated by SURVEYLOGISTIC regression analyses, which fit linear logistic regression models for discrete response survey data by using the maximum likelihood methods. Dependent variables were migraine and TTH in the models, and age and BMI were adjusted. All statistical analyses were conducted with SAS windows version 9.2 (SAS Institute Inc, Cary, North Carolina). The statistical significance level was set at *p* < 0.05.

### Ethics statement

All subjects gave informed consent for participation in this study. The conduct of this study was consistent with the International Conference on Harmonization’s ethical principles for medical research involving human subjects and the principles of the Declaration of Helsinki, and full compliance with privacy principles was maintained [15,16].

## Results

Our interviewers approached 4054 individuals and 1699 of them accepted the survey. Finally, 1507 subjects completed the survey. The distribution of SES status by gender of all participants did not differ from those of the total Korean population (Table 1). Among 1507 participants, the 1-year prevalence rates of migraine and tension-type headache were 8.7% [95% confidence interval (CI) 1.9-4.6%] and 29.1% (95% CI 25.7-32.5%) in women and 3.2% (95% CI 1.9-4.6%) and 32.5% (95% CI 29.1-35.9%) in men, respectively. The headache burden of migraine and TTH assessed by HIT-6 were 51.9 ± 1.08, 44.6 ± 0.49 in women and 51.8 ± 2.05, 43.5 ± 0.42 in men.

**Table 1:** Distribution of survey participants and of cases identified as migraine and tension-type headache

	**Survey participants, n (%)**	**Total Korean population, n (%)**	**p-value**^**b**^	**Survey participants, n (%)**		**Total Korean population, n (%)**		**p-value**^**b**^
				**Men**	**Women**	**Men**	**Women**	
Age								
19-29	241 (22.8^a^)	7,717,947 (22.2)	0.99	143 (51.5^a^)	98 (48.5^a^)	4,020,889 (52.1)	3,697,058 (47.9)	0.99
30-39	340 (23.5^a^)	8,349,487 (24.0)		150 (50.3^a^)	190 (49.7^a^)	4,206,218 (50.4)	4,143,269 (49.6)	0.99
40-49	418 (23.0^a^)	8,613,110 (24.8)		180 (50.4^a^)	238 (49.6^a^)	4,320,917 (50.2)	4,292,193 (49.8)	1.00
50-59	324 (19.8^a^)	6,167,505 (17.7)		168 (43.0^a^)	156 (57.0^a^)	3,052,099 (49.5)	3,115,406 (50.5)	0.36
60-69	184 (10.8^a^)	3,934,666 (11.3)		114 (53.0^a^)	70 (47.0^a^)	1,898,776 (48.3)	2,035,890 (51.7)	0.51
Size of residential area								
Large city	704 (46.7^a^)	16,776,771 (48.2)	0.89	354 (50.2^a^)	350 (49.8^a^)	8,314,969 (49.6)	8,461,802 (50.4)	0.93
Medium-to-small city	658 (43.7^a^)	15,164,345 (43.6)		329 (49.4^a^)	329 (50.6^a^)	7,475,219 (49.3)	7,689,126 (50.7)	0.99
Rural area	145 (9.6^a^)	2,841,599 (8.2)		72 (45.7^a^)	73 (54.3^a^)	1,487,149 (52.3)	1,354,450 (47.7)	0.35
Educational level								
Middle school or less	240 (15.9^a^)	6,291,149 (19.0)	0.84	105 (37.1^a^)	135 (62.9^a^)	2,419,857 (38.5)	3,871,292 (61.5)	0.84
High school	712 (47.2^a^)	14,530,056 (43.8)		339 (48.1^a^)	373 (55.5^a^)	7,499,067 (51.6)	7,030,989 (48.4)	0.46
College or more	555 (36.8^a^)	12,331,670 (37.2)		311 (55.5^a^)	244 (44.5^a^)	6,618,502 (53.7)	5,713,168 (46.3)	0.80
Total	1,507 (100.0^a^)	34,782,715 (100.0)				17,584,365 (49.6)	17,198,350 (50.4)	

^a^ Standardized prevalence.

^b^ Compare gender, age group, size of residential area, and educational level distributions between the sample of the present study and total population of Korea.

### Migraine and socioeconomic status

In all participants, multiple regression analysis revealed that living in medium-to-small city (OR 1.74, 95% CI 1.07-2.84) or rural areas (OR 2.61, 95% CI 1.30-5.27) related to a higher prevalence of migraine. Level of education and household income did not significantly influence prevalence of migraine (Table 2).

**Table 2:** One-year prevalence of migraine by educational level, district size, and income according to gender

**Characteristics**	**All participants**			**Women**			**Men**		
	**Prevalence,% (95% CI)**	**OR (95% CI)**	**p-value**	**Prevalence,% (95% CI)**	**OR (95% CI)**	**p-value**	**Prevalenc,% (95% CI)**	**OR (95% CI)**	**p-value**
Educational level									
Middle school or less	7.4 (3.9-10.9)	1.00		10.4 (5.3-15.5)	1.00		2.34 (0.0-5.6)	1.00	
High school	5.9 (4.1-7.7)	0.85 (0.44-1.65)	0.85	8.6 (5.7-11.7)	1.04 (0.43-2.50)	0.98	2.9 (1.0-4.8)	0.52 (0.08-3.49)	0.56
College or more	5.7 (3.8-7.5)	0.81 (0.39-1.65)	0.81	8.0 (4.8-11.3)	1.06 (0.35-3.22)	0.92	3.8 (1.6-6.0)	0.43 (0.05-3.51)	0.44
Size of district									
Large city	4.1 (2.6-5.5)	1.00		4.4 (2.3-6.6)	1.00		3.7 (1.7-5.8)	1.00	
Medium-to-small city	7.1 (5.1-9.0)	1.74 (1.07-2.84)	0.03	11.2 (7.8-14.7)	2.79 (1.40-5.54)	0.0004	2.8 (0.9-4.7)	0.82 (0.30-2.25)	0.64
Rural area	11.1 (5.8-16.3)	2.61 (1.30-5.27)	0.01	17.8 (9.0-26.5)	4.52 (1.85-11.02)	<0.0001	3.1 (0.0-7.5)	1.09 (0.23-5.29)	0.92
Household income (10,000 won/m)									
-199	7.1 (4.6-9.7)	1.00		11.7 (7.1-16.2)	1.00		2.7 (0.3-5.0)	1.00	
200-299	5.7 (3.4-7.9)	0.88 (0.47-1.64)	0.68	7.7 (4.1-11.3)	0.79 (0.36-1.74)	0.69	3.4 (0.8-6.0)	1.10 (0.29-4.26)	0.80
300-399	4.2 (2.1-6.3)	0.75 (0.37-1.49)	0.41	5.2 (2.0-8.4)	0.60 (0.23-1.56)	0.57	3.2 (0.4-6.0)	0.95 (0.22-4.10)	0.99
400-	7.5 (4.6-10.4)	1.38 (0.71-2.68)	0.34	10.9 (5.7-16.1)	1.40 (0.57-3.44)	0.24	3.2 (0.6-5.9)	1.10 (0.24-4.95)	0.78
BMI									
< 25	6.3 (4.9-7.7)	1.00		9.0 (6.7-11.3)	1.00		3.1 (1.6-4.5)	1.00	
≥ 25	4.2 (2.0-6.5)	0.63 (0.35-1.15)	0.13	5.3 (1.0-9.7)	0.91 (0.43-1.97)	0.82	3.7 (1.1-6.3)	1.36 (0.53-3.49)	0.52
Total	6.0 (4.8-7.2)			8.7 (1.9-4.6)			3.2 (1.9-4.6)		

*Note:* Multiple logistic regression analysis adjusted for all other determinants, age and BMI. BMI: Body mass index; CI: confidence interval; OR: odds ratio.

In women, the prevalence rates of migraine were 17.8%, 11.2% and 4.4% in rural areas, medium-to-small cities, and large cities in women, respectively. Multiple regression analysis revealed that living in medium-to-small city (OR 2.79, 95% CI 1.40-5.54) or rural areas (OR 4.52, 95% CI 1.85-11.02) related to a higher prevalence of migraine. Level of education and household income did not significantly influence prevalence of migraine (Table 2).

In men, the prevalence rates of migraine did not differ according to variables of socioeconomic status and multiple regression analysis revealed that no socioeconomic variable influenced the prevalence of migraine (Table 2).

### TTH and socioeconomic status

In all participants, multiple regression analysis revealed that college-level education (OR 0.60, 95% CI 0.43–0.92) and living in rural areas (OR 0.56, 95% CI 0.36–0.87) was related to lower prevalence of TTH, while household income did not show any significant influence on prevalence (Table 3).

**Table 3:** One-year prevalence of tension-type headache by educational level, district size, and income according to gender

**Characteristics**	**All participants**			**Women**			**Men**		
	**Prevalence,% (95% CI)**	**OR (95% CI)**	**p-value**	**Prevalence,% (95% CI)**	**OR (95% CI)**	**p-value**	**Prevalenc,% (95% CI)**	**OR (95% CI)**	**p-value**
Educational level									
Middle school or less	33.8 (27.6-40.1)	1.00		38.8 (30.1-47.0)	1.00		25.7 (15.8-35.0)	1.00	
High school	34.3 (30.7-38.0)	0.96 (0.68-1.35)	0.80	31.2 (26.3-36.1)	0.66 (0.38-1.28)	0.12	37.2 (32.4-43.1)	1.41 (0.80-2.51)	0.29
College or more	26.0 (22.6-29.5)	0.60 (0.43-0.92)	0.02	21.5 (16.7-26.4)	0.37 (0.18-0.74)	0.003	28.9 (24.6-34.3)	0.88 (0.45-1.72)	0.64
Size of district									
Large city	31.6 (28.2-35.0)	1.00		31.9 (27.0-36.8)	1.00		31.1 (26.4-36.1)	1.00	
Medium-to-small city	31.9 (28.4-35.5)	0.99 (0.78-1.25)	0.90	29.0 (24.1- 34.0)	0.77 (0.54-1.10)	0.13	34.6 (29.4-39.9)	1.18 (0.85-1.66)	0.24
Rural area	22.4 (15.4-29.3)	0.56 (0.36-0.87)	0.01	16.3 (7.8-24.8)	0.29 (0.15-0.58)	0.0008	2 (17.9-40.8)	0.99 (0.54-1.81)	0.85
Household income (10,000 won/m)									
-199	32.4 (27.7-37.0)	1.00		33.9 (27.1-40.6)	1.00		30.9 (24.5-37.3)	1.00	
200-299	29.4 (25.0-33.9)	0.93 (0.67-1.28)	0.64	24.7 (18.9-30.6)	0.70 (0.42-1.15)	0.22	34.7 (27.9-41.5)	1.14 (0.72-1.79)	0.59
300-399	31.2 (26.4-36.1)	0.99 (0.71-1.38)	0.95	28.4 (21.9-34.9)	0.78 (0.46-1.32)	0.21	34.0 (26.8-41.1)	1.12 (0.70-1.80)	0.45
400-	30.7 (25.6-35.8)	1.07 (0.75-1.53)	0.71	30.1 (22.8-37.3)	0.98 (0.56-1.72)	0.92	31.1 (23.8-38.4)	1.08 (0.64-1.81)	0.89
BMI									
< 25	29.5 (26.9-32.1)	1.00		27.6 (24.1-31.1)	1.00		31.7 (27.8-35.6)	1.00	
≥ 25	36.3 (30.9-41.6)	1.31 (1.00-1.71)	0.05	39.1 (29.8-48.4)	1.24 (0.80-1.93)	0.34	34.9 (28.3-41.5)	1.16 (0.82-1.65)	0.40
Total	30.8 (28.5-33.1)			29.1 (25.7-32.5)			32.5 (29.1-35.9)		

*Note:* Multiple logistic regression analysis adjusted for all other determinants, age, and BMI. BMI: Body mass index; CI: codence interval; OR: odds ratio.

In women, the prevalence rates of TTH were 38.8%, 31.2% and 21.5% among middle school or less graduates, high school graduates, and college or more graduates, respectively. According to residential area, the prevalence rates were 16.3%, 29.0% and 31.9% in rural areas, medium-to-small cities, and large cities. Multiple regression analysis revealed that college-level education (OR 0.37, 95% CI 0.18–0.74) and living in rural areas (OR 0.29, 95% CI 0.15–0.58) was related to lower prevalence of TTH, while household income did not show any significant influence on prevalence (Table 3).

In men, the prevalence rates of TTH did not differ according to variables of socioeconomic status and multiple regression analysis revealed that no socioeconomic variable influenced the prevalence of migraine.

## Discussion

The strong points of this study were its assessment of the influence of SES in an Asian population and the use of the ICHD-II to diagnose migraine and TTH. In this nationwide, population-based study, the influence of SES on the prevalence rates of migraine and TTH was prominent in women. In women, living in rural areas was associated with increased risk of migraine, while living in rural areas and having college-level of education was related to decreased risk of TTH. We also found that the influence of SES on primary headache disorders may not be evident in men and differed according to the subtype of headache. This result suggests that gender differences in migraine or TTH may be partly related to the gender-specific influence of socioeconomic status.

Because of the social inequality between men and women, the collection of gender-disaggregated data is recommended [17]. However, a few studies have investigated the gender-specific influences of SES on migraine or TTH. In a study in Denmark, low level of education was associated with increased risk of migraine in women (but showed no influence in men) [4]. In a *Women’s Health* study, women with low SES showed increased risk of all forms of headache, including migraine [5]*.* Interestingly, prevalence of frequent and chronic headache was associated with low individual income in men (but not in women) in a Norwegian study [6], and income adequacy in men and dwelling type in women have been identified as significant determinants of self-reported poor health in Canada [18]. A few studies have examined the influence of SES in Asian populations, so this study offers a glimpse into those data in Asia [11,19]. Although Korea is an OECD country that was ranked 11^th^ among 187 countries in the United Nations Development Program’s 2011 Gender Inequality Index [20], women had a higher chance to have lower level of eduction (Table 1, p < 0.001 by an additional chi-square analysis for all participants). The social inequality between men and women may be related to women’s increased risk of migraine or TTH in Korea.

The observed influence of SES in Korea was somewhat different from that observed in previous studies. First, the beneficial influence of education was on TTH but not evident on migraine in this study. Lower level of education was related to increased risk of migraine in studies in the US and Denmark [4,5] and was related to an increased risk of chronic headache in a study in Norway [6], but showed no association in a study from Canada [21]. Although we could not exclude the possibility of a chance, this international variation may be partly related to the low prevalence of migraines in Koreas and may be partly rooted in genetic or cultural differences [22]. Contrary to this study, low level of education was shown to decrease the risk of episodic TTH in previous studies in the US, Brazil, and Croatia [3,23,24]. Generally speaking, higher level of education is associated with less pain and disability, which may reflect better pain coping strategies [25]; nevertheless, the economic returns of education vary by gender, ethnicity, and era [1,25,26]. It is uncertain whether a higher level of education imparts better pain coping strategies for TTH in Korea and Norway, while increasing stressful responsibility in other countries.

For women, living in rural areas increased the risk of migraine (but not TTH) in this study. A similar trend was reported in a study from the Republic of Georgia, but the opposite result was reported in a study from Croatia [24,27]. Although the interaction between living areas and level of education or household incomes was not included in purpose of this study, participants who living in rural areas had lower frequency of college-level education than those living in medium-to-small city or big city (18.8% vs. 38.8%, p-value < 0.001 by Mantel-Haenszel analysis) and had lower household income than those living in medium-to-small city or big city (p < 0.001 by Mantel-Haenszel analysis). Therefore, the feasibility and the interaction of living areas as a proxy of SES should be evaluated in future studies.

Household income was not related to the prevalence of migraine or TTH in this study, but an association between low income and increased risk of migraine or TTH has been consistently reported [6,7,27]. Household income is a reasonable index of SES, and individual income may serve to better assess the influence of SES on headache disorders according to their gender [6].

This study has some limitations. First, the number of participants was relatively small, and there were especially few men who experienced migraines. Although evaluation of headache disorder by ICHD-II criteria in large populations may not be easy, the limitations associated with the small sample size should be taken into account in the interpretation of these results. However, the number of men with TTH was not small, so the result seen in this study is likely not an incidental finding. Second, we did not use a composite index or analyze occupation as a proxy of SES, it might be helpful in the future to use a composite index [5]. Third, we did not evaluate the headache frequency or medication overuse as parameters of headache [28]. Headache burden was related to headache frequency, so this subject might be evaluated [6]. Finally, this study is a cross-sectional study, so the directionality of the associations is uncertain. The higher prevalence of migraine and any headaches in children with low SES suggests social causation [29]; however, the socioeconomic impact of migraine is not so small, so bidirectional influence is possible [30].

## Conclusions

Several indices of SES (e.g., district size of residential area and level of education) were related to risk of migraine and TTH, especially in women. Therefore, the influence of SES should be evaluated separately by gender.

## Competing interests

The authors declare that they have no competing interests.

## Authors’ contributions

CSJ and CMK carried out the design of the study, performed the statistical analysis, draft the manuscript. KDW participated in its design of the table and helped to draft the manuscript. KBK, KJM, and LKS participated in its design of the study and draft the manuscript. JTW carried out the statistical analysis and participated in its design of the table. PJW helped to draft the manuscript. All authors read and approved the final manuscript.
